# Differential expression of histamine receptors in the bladder wall tissues of patients with bladder pain syndrome/interstitial cystitis – significance in the responsiveness to antihistamine treatment and disease symptoms

**DOI:** 10.1186/s12894-019-0548-3

**Published:** 2019-11-12

**Authors:** Hui Shan, Er-Wei Zhang, Peng Zhang, Xiao-Dong Zhang, Ning Zhang, Peng Du, Yong Yang

**Affiliations:** 1 Department of Urology, Beijing Chaoyang Hospital, Capital Medical University, No.8 Gongti South Road, Beijing, 100020 China; 2 Department of Urology, The First Affiliated Hospital of Zhengzhou University, Zhengzhou, China; 3 Department of Urology, China Meitan General Hospital, Beijing, China; 4 Department of Urology, Beijing Cancer Hospital, Beijing, China

**Keywords:** Bladder pain syndrome, Interstitial cystitis, Histamine receptors, Anti-histamine bladder

## Abstract

**Background:**

Activation of mast cells plays an important role in the pathogenesis of bladder pain syndrome/interstitial cystitis (BPS/IC). Histamine, a mast cell-derived mediators, induced inflammation and hypersensitivity of the bladder. The present study investigated the expressions of histamine receptors in the bladder wall tissues of patients with BPS/IC, and its association with the effectiveness of antihistamine therapy and disease symptoms.

**Methods:**

Bladder tissues were collected from 69 BPS/IC patients and 10 control female patients. The expression of H3R in BPS/IC was further examined in an independent cohort of 10 female patients with BPS/IC and another 10 age-matched female patients. Immunohistochemistry, Western blotting, and quantitative RT-PCR were performed to quantify the expressions of histamine receptors. Statistical analyses of the correlation of histamine receptor expression with antihistamine therapy outcome and severity of disease symptoms were also performed.

**Results:**

The expression of four histamine receptors was significantly elevated in BPS/IC (H1R, *P* < 0.001; H2R, *P* = 0.031; H3R, *P* = 0.008; H4R, *P* = 0.048). Western blotting revealed that H3R were significantly reduced in the patients, whereas the mRNA levels of H3R were significantly increased. The patients were further divided into antihistamine responders (*n* = 38) and nonresponders (*n* = 22). No significant correlation was found in the expression of histamine receptors between responder and nonresponder groups. However, significant correlations between OLS and H1R (*P* = 0.003) and H3R (*P* = 0.045) were found.

**Conclusion:**

The present study showed that expression of all the 4 histamine receptors were elevated in BPS/IC. There were no statistical significant correlations between the expression levels of the four different histamine receptors and the treatment outcome of antihistamine therapy (amtitriptyline or cimetidine).

## Background

Bladder pain syndrome/Interstitial cystitis (BPS/IC), or bladder pain syndrome, is a complex bladder dysfunction characteristized by chronic (> 6 months) urinary bladder pain or discomfort, accompanied by other urinary symptom such as persistent urge to void [1]. BPS/IC can contribute to chronic pelvic pain and poor quality of life, which is now recognized as a serious medical condition. The disease occurs mostly in young and middle-aged women, with no known etiology. A survey conducted in US indicated that 575 in every 100,000 women had IC [2], and the prevalence rate of self-reported BPS/IC among community-dwelling adult women was around 4% [3].

Several etiologies have been proposed, including abnormalities in urine, infection, inflammation resulted from autoimmunity, activation of mast cells, neurogenic inflammation, and disturbance in permeability of the bladder wall [4]. Evidence from in vitro and clinical studies suggested that mast cells play a critical role in the pathogenesis and pathophysiology of BPS/IC [5, 6]. Upon the activation of mast cells, histamine is released; and the binding of histamine to the receptors on bladder wall could induce inflammation and hypersensitivity of the bladder [7, 8]. Treatments of BPS/IC include behavioral therapy, mucosal protection, histamine receptor antagonism, analgesia, and surgical treatment, whereas the most common interventions include bladder mucosal protective agents and anti-histamines [9].

There are four subtypes of histamine receptors: H1R, H2R, H3R, and H4R. It has been confirmed that the expression of all the four subtypes of histamine receptors were found in BPS/IC animal model, in which the receptors were expressed mainly in bladder epithelial cells. Significant differences were found in the expression of H1R, H2R, and H3R before and after BPS/IC [10]. However, it remains unclear on the pattern of expressions of the four histamine receptors in patients with IC. Also, it is not known whether there is an association between histamine receptor expression and efficacy of antihistamines.

In the current study, we analyze the correlation between expressions of histamine receptors and the severity of BPS/IC (as assessed by OLS score), as well as the effectiveness of antihistamine therapy.

## Methods

### Study population

The exploration study included the use of bladder tissues from 69 patients admitted to Beijing Chaoyang Hospital (Beijing, China) from 2005 to 2009 for treatment of interstitial cystitis. The diagnosis of BPS/IC was based on the guidelines published by the National Institute of Diabetes and Digestive and Kidney Diseases. Tissues were collected by biopsy forcep and were retrospectively analyzed. The study also collected bladder tissues as the control samples from 10 female patients with other urinary complications but no complaints of bladder pain. For the validation study, bladder tissues were collected from 10 patients with BPS/IC and 10 age-matched females with no BPS/IC but receiving surgical treatment for stress urinary incontinence. Tissues were collected by the use of cystoscopy from the trigone, and from the front, back, left and right walls of the bladder. The study design was approved by the ethnic committee of our hospital, with informed consents obtained from all the participants.

### Antibodies and reagents

Antibodies against human histamine H1, H2, H3, and H4 receptors were purchased from Santa Cruz (Dallas, TX, USA). Rabbit polyclonal antibody antibody against human histamine H3 receptor was obtained from LifeSpan Bioscience (Seattle, WA, USA). Chemiluminescent HRP substrate was from Chemicon (Temecula, CA, USA). Total RNA was isolated using RNA extraction kit from Ambion (Carlsbad, CA, USA), and was then transcribed and amplified using GoTaq® 2-Step RT-qPCR System (Promega, Madison, WI, USA).

### Immunohistochemistry and quantitative image analysis

Formalin-fixed paraffin-embedded bladder tissues were sectioned, dewaxed, and stained with antibodies against human histamine receptors at 4 °C overnight. After the incubation with secondary antibody and washing, the reactivity signal was developed using DAB. Under microscopic observations (a magnification, 400x) three different fields were captured for signal quantitation. Image acquisition software Image Pro-Plus 6.0 was employed to measure the integral optical density (IOD) and area of each selected microscopic field. The mean density (MD) was then calculated as IOD/avea. Microscopic fields with MD values < 0.001 were determined as negative for human histamine receptors.

### Western blotting and quantitative RT-PCR for H3R

The protein and gene expression of H3R in the validation cohort was examined using western blotting and quantitative RT-PCR, respectively. For western blotting, tissue homogenates were resolved by electrophoresis, transferred to PVDF membranes and probed with rabbit polyclonal antibody against H3R. Semi-quantitation of the band intensity of H3R was then performed using Quantity One software. For quantitative RT-PCR, H3R gene was amplified by STRATA3000 system with the level determined using -2^ΔΔCT^ approach.

### Statistical analysis

Data analysis was done using statistical software SPSS version 19.0. Measurement data were presented as mean ± standard deviation, and were compared between groups using *t*-test. Statistically different comparison was indicated by *P* value <0.05.

## Results

### Differential expression of human histamine receptors in BPS/IC bladder tissues

**Fig. 1:**
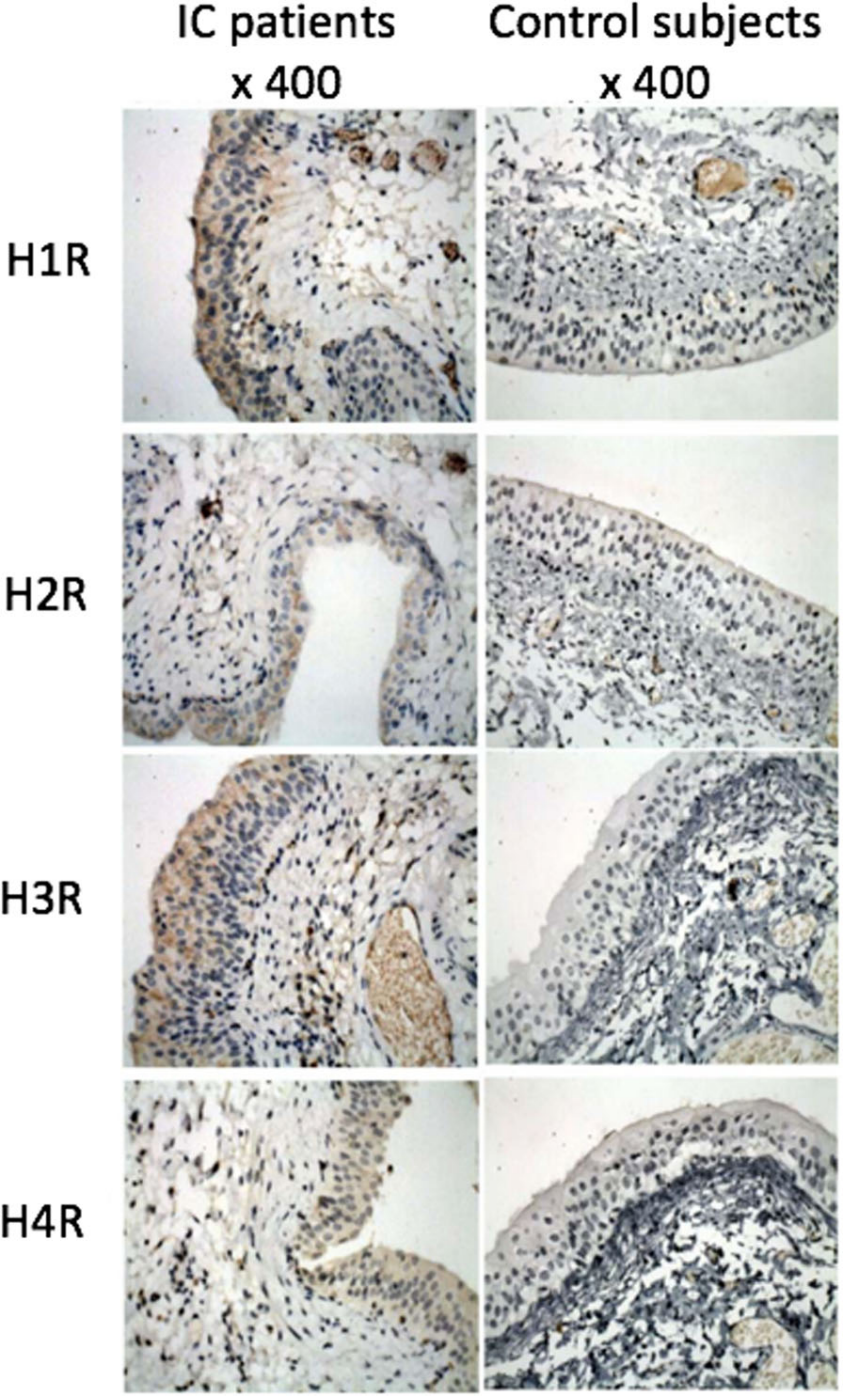
Expression of the histamine receptors in bladder tissues was studied using immunohistochemistry. The receptors were mainly expressed in the mucosa of bladder, the interstitial blood vessels and the detrusor muscles. Shown are the representative set of images with magnification of 400x

The expression of four different histamine receptors in the bladder tissues of 69 patients with BPS/IC was examined using immunohistochemistry (Fig. 1). The receptors were mainly expressed in the mucosa of bladder, the interstitial blood vessels and the detrusor muscles. The IHC signals were quantified (Table 1). Compared to the bladder tissues of subjects with no BPS/IC (*n* = 10), the expression of four histamine receptors was significantly elevated in BPS/IC (H1R, *P* < 0.001; H2R, *P* = 0.031; H3R, *P* = 0.008; H4R, *P* = 0.048).

**Table 1:** Expression of histamine receptors in IC patients and in control subjects

Groups	Number of cases	H1R	H2R	H3R	H4R
IC patients	69	0.1389 ± 0.05115	0.0861 ± 0.01646	0.1124 ± 0.04665	0.1007 ± 0.02212
Control Subjects	10	0.0395 ± 0.00361	0.0633 ± 0.00809	0.0842 ± 0.01632	0.0576 ± 0.00982
*P-* value		0.000	0.031	0.008	0.048

### H3R expression in an independent IC cohort

**Fig. 2:**
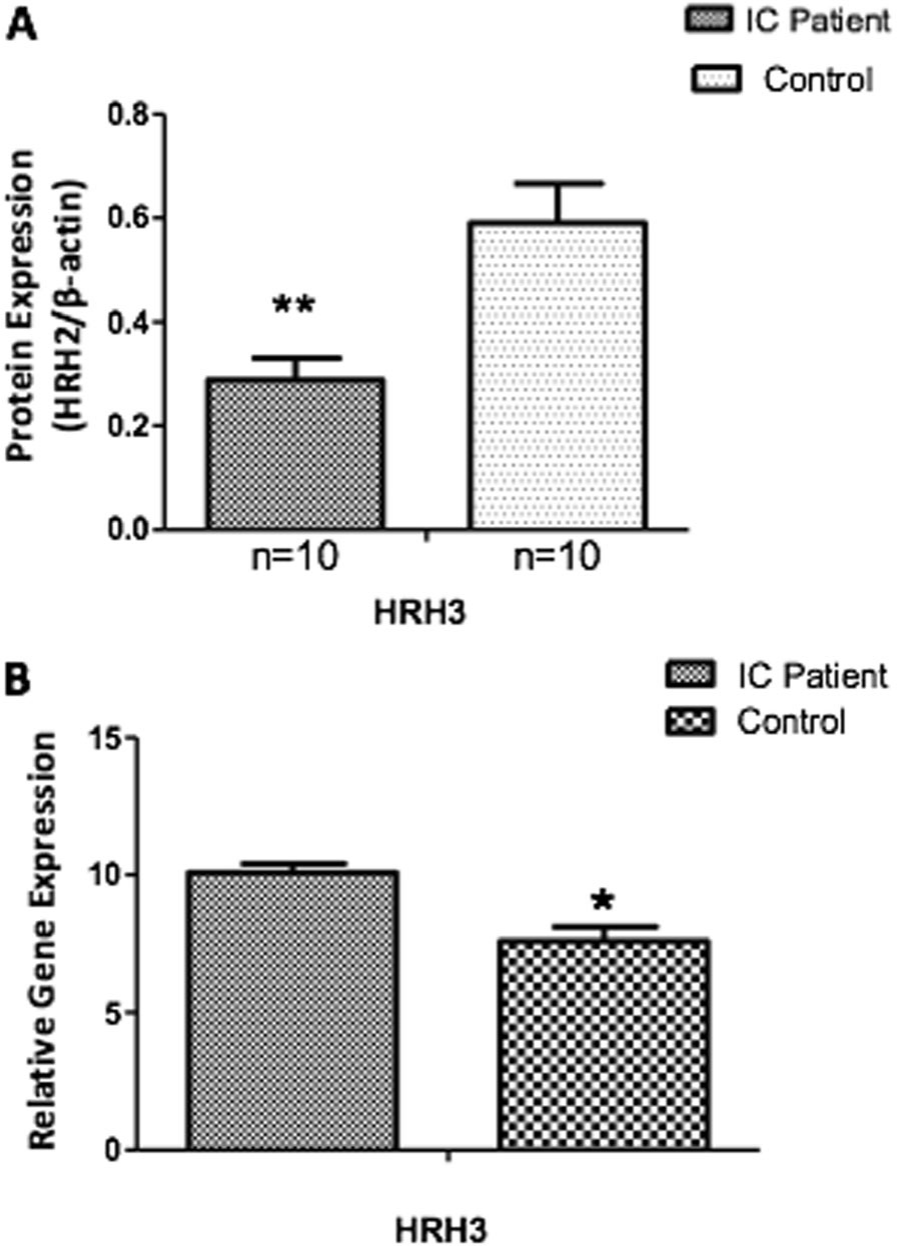
Validation of H3R expression in an independent cohort of patients with IC and age-matched female patients with stress urinary incontinence. **a** Western blotting showed that H3R protein level in patients with IC (*n* = 10) was found down regulated when compared with those without IC (*n* = 10). **b** The gene expression of H3R in IC and stress urinary incontinence was examined using real-time PCR, showing the expression was significantly elevated in IC. Shown are the representative set of data of three independent measurements

The expression of H3R in BPS/IC was further examined in an independent cohort of 10 female patients with BPS/IC and another 10 age-matched female patients receiving surgical treatment for stress urinary incontinence. The protein levels of H3R in the tissues were examined using western blotting. Western blotting revealed that H3R protein levels were significantly reduced in the bladder tissues of patients with BPS/IC comparing to those without BPS/IC (*P* < 0.01) (Fig. 2a). The gene expression of H3R in the tissues was studied using quantitative real-time PCR. Result demonstrated that the mRNA transcript levels of H3R were significantly increased in IC tissues when compared to those without IC (Fig. 2b).

### Correlation of histamine receptor expression with antihistamine therapy outcome and OLS

After showing the elevation of four different histamine receptors in BPS/IC bladder tissues, we next investigated whether these expression would correlate the outcome of antihistamine treatment. Patients in this study received anti-histamine (amitriptyline or cimetidine) as first-line treatment, and those demonstrated an improvement of clinical symptoms (e.g. pain, urgency, and frequency) were classified as responders. We examined the histamine receptor expression in antihistamine responders (*n* = 38) and nonresponders (*n* = 22). In the responder group, there were 9 male and 29 female patients aged 25 to 80 years (average age, 50.59 ± 14.29 years), while in the nonresponder group, there were 2 male and 20 female patients aged 24 to 70 years (average age, 40.06 ± 12.63 years). Our analysis showed that the expression of histamine receptors between responder and nonresponder groups was statistically not significant (H1R, *P* = 0.362; H2R, *P* = 0.082; H3R, *P* = 0.869; H4R, *P* = 0.292) (Table 2).

**Table 2:** Expression of histamine receptors in responders and nonresponders

Groups	Number of cases	H1R	H2R	H3R	H4R
Responders	39	0.1364 ± 0.04775	0.0863 ± 0.01820	0.1127 ± 0.04903	0.1029 ± 0.02008
Non-responders	21	0.1407 ± 0.05580	0.0851 ± 0.01303	0.1201 ± 0.04578	0.0938 ± 0.02655
*P*-value		0.362	0.082	0.869	0.292

The correlation between histamine receptor expression and symptom severity was also evaluated. The symptoms of BPS/IC were assessed using the O’Leary-Sant (OLS) questionnaire [11]. The mean OLS of the cohort was 29.39 ± 5.273. The correlations between the expression of individual receptor and OLS score were listed in Table 3. Significant correlations with OLS were found in H1R (*P* = 0.003) and H3R (*P* = 0.045).

**Table 3:** Correlation of histamine receptor expression and OLS

		H1R	H2R	H3R	H4R
OLS	Correlation coefficient	0.360	0.026	0.244	−0.157
	*P*-value	0.003	0.837	0.045	0.202

## Discussion

The present study examined the differential expression of 4 histamine receptors (H1R, H2R, H3R, and H4R) in the bladder tissues of patients with BPS/IC, showing all the receptors were significantly elevated in BPS/IC. Our analysis also suggested that although histamine receptors are biologically relevant to BPS/IC pathogenesis, there were no significant correlations between the expression levels of the four different histamine receptors and the treatment outcome of antihistamine therapy.

Despite the accumulated studies over the decades, the etiology of BPS/IC have yet to be fully studied [12]. Different hypothesized etiologies have been proposed, including abnormalities in urine, infection, inflammation resulted from autoimmunity, activation of mast cells, neurogenic inflammation, and disturbance in permeability of the bladder wall [4]. Epithelial antiproliferative factor plays a key role in inhibiting the growth of endothelial cell of the bladder. Baltimore et al. recently identified that the mutation of FZB8 gene would affect the normal functions of epithelial antiproliferative factor, disrupting the integrity of bladder mucosa, resulting in an increase in the permeability of the bladder [4, 7, 13]. Nevertheless, none of these hypotheses can fully explain the diverse clinical manifestations and differential drug responses among patients with BPS/IC.

The understanding on the pathophysiology is relatively clear comparing to the disease etiology. It has been widely accepted that BPS/IC is resulted from the chronic inflammation of the entire bladder wall, in which mast cells represent one of the key players. In patients with BPS/IC activated mast cells were found in the bladder of the patients [6]. By releasing histamine that binds to the receptors on bladder wall, the mast cells can induce inflammation and hypersensitivity of the bladder [7, 8]. In accordance with other previous studies, we herein demonstrated that all of the four histamine receptors were over expressed in the bladder wall of BPS/IC. Moreover, our examination clearly depicted the localisation of IHC signals mainly in the mucosa of bladder, with a lesser extent of reactivity could be detected in the interstitial blood vessels and the detrusor muscles. There results likely implicated that the mucosa of the bladder would be the major target of histamine released from the infiltrated mast cells, in turn, the major pathogenic sites of BPS/IC. Our study has therefore provided further evidences to support the hypothesis that BPS/IC would be resulted from the dysfunction of the bladder mucosa, and to strengthen the rationale of using antihistamine in the treatment of BPS/IC.

Histamine modulates diverse immunological responses through its activation on different histamine receptors. All the four histamine receptors are G-protein coupled receptors expressing in diverse tissues with distinct functions [14, 15]. H1R mediates inflammatory reaction [16], while H2R shows inhibitory effects on the apoptosis of mononuclear cells [17]. H3R mainly controls the inflammatory reactions by modulating the release of pro-inflammatory peptides [18], while H4R promotes the acute inflammatory responses through its enhancement on the migration of eosinophils and mast cells [19]. The pathogenic roles of H1 and H2 receptors in BPS/IC have been reported. H3R and H4R are relatively new histamine receptors, therefore, their roles in IC have remained largely unclear. In this study, we showed that the expressions of both H3R and H4R were increased in IC. The total protein of H3R, however, was reduced in an independent cohort of BPS/IC using Western blotting, suggesting a post-transcriptional modification of H3R.

We have not found significant differences in the expression of histamine receptors between responders and nonresponders. There were several possible explanations for the insignificant results: 1) the small sample size; 2) the expressions of all the four histamine receptors were relatively high in patients, however, only H1R and H2R antagonists were applied; 3) a drug treatment duration of 3 months is needed for antihistamine to achieve therapeutic effects. Some patients might fail to adhere to the medication schedule, thus the treatment outcome became sub-optimal. In future studies, H3R and H4R antagonists may be added to achieve sufficient inhibition of histamine receptors, and the correlation between the cystoscopic findings and expression of histamine receptors/ clinical outcome can be investigated. Further, H3R was significantly correlated with OLS, implicating the potential role of H3R in disease progression of BPS/IC.

## Conclusion

In conclusion, the present study reported the increased expression of H1R, H2R, H3R, and H4R in BPS/IC. The expressions of the receptors were not statistically correlated with the outcome of antihistamine therapy (amtitriptyline or cimetidine); however, H1R and H3R were related to the disease severity as assessed by OLS, implicated the potential role of H3R in disease progression.

## Data Availability

The datasets generated and analyzed during the current study are available from the corresponding author on reasonable request.

## Abbreviation

BPS/IC: Bladder pain syndrome/interstitial cystitis

## References

[1] van de Merwe JP (2008). Diagnostic criteria, classification, and nomenclature for painful bladder syndrome/interstitial cystitis: an ESSIC proposal. Eur Urol.

[2] Rosenberg MT, Hazzard M (2005). Prevalence of interstitial cystitis symptoms in women: a population based study in the primary care office. J Urol.

[3] Ibrahim IA (2007). Prevalence of self-reported interstitial cystitis (IC) and interstitial-cystitis-like symptoms among adult women in the community. Int Urol Nephrol.

[4] Teichman JM, Moldwin R (2007). The role of the bladder surface in interstitial cystitis/painful bladder syndrome. Can J Urol.

[5] Sant GR (2007). The mast cell in interstitial cystitis: role in pathophysiology and pathogenesis. Urology.

[6] Theoharides TC, Kempuraj D, Sant GR (2001). Mast cell involvement in interstitial cystitis: a review of human and experimental evidence. Urology.

[7] Jutel M, Blaser K, Akdis CA (2006). Histamine receptors in immune regulation and allergen-specific immunotherapy. Immunol Allergy Clin N Am.

[8] Thurmond RL, Gelfand EW, Dunford PJ (2008). The role of histamine H1 and H4 receptors in allergic inflammation: the search for new antihistamines. Nature reviews. Drug Des Discov.

[9] Theoharides TC (2008). Interstitial cystitis: bladder pain and beyond. Expert Opin Pharmacother.

[10] Neuhaus J (2006). Histamine receptors in human detrusor smooth muscle cells: physiological properties and immunohistochemical representation of subtypes. World J Urol.

[11] O'Leary MP (1997). The interstitial cystitis symptom index and problem index. Urology.

[12] Toft BR, Nordling J (2006). Recent developments of intravesical therapy of painful bladder syndrome/interstitial cystitis: a review. Curr Opin Urol.

[13] Keay S (2004). Antiproliferative factor, heparin-binding epidermal growth factor-like growth factor, and epidermal growth factor in men with interstitial cystitis versus chronic pelvic pain syndrome. Urology.

[14] Breunig E (2007). Histamine excites neurones in the human submucous plexus through activation of H1, H2, H3 and H4 receptors. J Physiol.

[15] Strakhova MI (2009). Localization of histamine H4 receptors in the central nervous system of human and rat. Brain Res.

[16] Akdis CA, Blaser K (2003). Histamine in the immune regulation of allergic inflammation. J Allergy Clin Immunol.

[17] Jutel M, Akdis CA (2007). Histamine as an immune modulator in chronic inflammatory responses. Clin Exp Allergy.

[18] Cannon KE, Leurs R, Hough LB (2007). Activation of peripheral and spinal histamine H3 receptors inhibits formalin-induced inflammation and nociception, respectively. Pharmacol Biochem Behav.

[19] Coruzzi G (2007). Antiinflammatory and antinociceptive effects of the selective histamine H4-receptor antagonists JNJ7777120 and VUF6002 in a rat model of carrageenan-induced acute inflammation. Eur J Pharmacol.

